# Prevalence of intimate partner violence against women and associated factors in Ethiopia

**DOI:** 10.1186/s12905-020-0892-1

**Published:** 2020-02-07

**Authors:** Ayele Gebeyehu Chernet, Kebadu Tadesse Cherie

**Affiliations:** Department of Statistics, College of Natural and Computational Sciences, Wolkite University, Wolkite, Ethiopia

**Keywords:** Intimate partner violence, Prevalence, Logistic regression, Ethiopia

## Abstract

**Background:**

Violence against women is a major public health problem that affects the physical, sexual, mental, and social wellbeing of more than one third of all women globally. Violence against women in Ethiopia is widely acknowledged to be of great concern from human rights, economic and health perspective. The aim of this study was to assess the prevalence of intimate partner violence (IPV) against women and associated factors in Ethiopia.

**Method:**

The data was obtained from 2016 Ethiopia Demographic and Health Survey which is the fourth survey conducted in Ethiopia as part of the worldwide project. The sample was selected using a stratified; two-stage cluster sampling design and the data was analyzed using logistic regression model.

**Result:**

A total of 4714 ever-married women in reproductive age who reported their experience of spousal violence were considered from nine regional states and two city administratives. Over 30% of study participants were subjected to IPV. Living in rural areas, divorced, primary and secondary education, 25–39 years old, being poor are found to be predictors of IPV against women in Ethiopia.

**Conclusion:**

The prevalence of IPV was found high in Ethiopia and government and any concerned bodies should design appropriate strategy and work hard to tackle the problem. There is a need of giving special attention for women living in rural area, women from poor family and 25–39 years old women to decrease the burden of IPV.

## Background

Violence against women is a main public health problem that affects the physical, sexual, mental, and social wellbeing of more than one third of all women worldwide [1]. It is the deliberate and often repetitive physical, sexual, psychological, or economic abuse. The most common form of domestic violence is the ones committed in contradiction of women by their intimate partner [2, 3]. Despite the Universal Declaration of the Human Rights, as all people begin to be recognized regardless of age, sex, race, color, language, religion, or any other factors, women have continued to suffer from domestic violence and discrimination in their homes [4].

Research conducted by WHO on domestic violence revealed that intimate partner violence (IPV) is the most common form of violence in women’s lives and showed that women were at higher risk of violence at homes than in the streets and this has serious influences on women’s health [5]. Sexually and physically abused women by intimate partners have a risk of 50 to 70% to be affected by gynecological, central nervous system, and stress related problems [6]. The World Bank has documented gender based violence as a heavy health problem for women aged 15 to 44 similar to the risk pretended by HIV, tuberculosis, infection during childbirth, cancer and heart disease [7].

IPV refers to behavior by an intimate partner that causes physical, sexual or psychological devastation, including actions of physical violence, sexual intimidation, and emotional abuse and controlling behaviors [8]. Many researches have verified a high prevalence of violence against women by intimate partners globally and the associated physical and mental health problems [9, 10].

According to multi-country study on women’s health and domestic violence against women, the lifetime prevalence of physical, sexual, or both physical and sexual violence ranges from 15% (Japan) to 71% (Ethiopia). Nearly one half (49%) of ever-married women faced physical violence, 59% of them experienced sexual violence, 71% of them had one or the other form of violence, or both, over their life time. About 35% of all ever-married women experienced at least one severe form of violence by a partner [11]. A cross sectional study conducted in Nigeria showed that almost one in four (21.5%) ever-married women faced IPV at some point in their lives [12].

According to research conducted in Gonder referral Hospital, the overall prevalence of domestic violence among pregnant women was estimated to be 58.7% with emotional violence being the most common (57.8%), followed by physical violence (32.2%), and sexual violence (7.6%). This research also showed that house wives, women with no salary of their own, partners’ daily use of alcohol, and women who disobeyed their partner were found to be positively and significantly associated with domestic violence during pregnancy [13].

In Ethiopia, violence against women is widely acknowledged to be of great concern, not just from a human rights perspective, but also from an economic and health viewpoint. The government of Ethiopia reviewed its family law in 2000, its criminal law and constitution in 2005, to protect and assurance the rights of women and children, and to promote gender equality and equity. IPV affects all the domains of women’s lives such as self-esteem, productivity, autonomy, capacity to care for themselves and their children, ability to participate in social activities, and even death [11, 14]. Despite the international emphasis to reduce violence against women, the size of IPV is very high in Ethiopia. Thus, the aim of this study was to assess the prevalence of IPV against ever-married women and associated factors in Ethiopia.

## Methods

### Source of data and study design

The data was obtained from 2016 EDHS, which was taken from Central Statistical Agency (CSA). It is the fourth survey conducted in Ethiopia as part of the worldwide project. The 2016 EDHS sample was stratified and selected in two stages. Each region was stratified into urban and rural areas, yielding 21 sampling strata. Samples of Enumeration Areas (EAs) were selected independently in each stratum in two stages. Implicit stratification and proportional allocation were used at each of the lower administrative levels by sorting the sampling frame within each sampling stratum before sample selection, according to administrative units in different levels, and by using a probability proportional to size selection at the first stage of sampling.

In the first stage, a total of 645 EAs (202 EAs in urban areas and 443 EAs in rural areas) were selected with probability proportional to the EA size (based on the 2007 Population and House Census) and with independent selection in each sampling stratum. The resulting lists of households served as a sampling frame for the selection of households in the second stage. Some of the selected EAs were large, with more than 300 households. To minimize the task of household listing, each large EA selected for the 2016 EDHS was segmented. Only one segment was selected for the survey, with probability proportional to the segment size. Household listing was conducted only in the selected segment, that is, a 2016 EDHS cluster is either an EA or a segment of an EA.

In the second stage of selection, a fixed number of 28 households per cluster were selected with an equal probability systematic selection from the newly created household listing. Information was collected on IPV for ever-married women age 15–49 who reported their experience of spousal emotional, physical, and sexual violence. After excluding missing values, a total of 4714 ever-married women in reproductive age who reported their experience of spousal violence were considered in this study [15].

### Outcome variable

The response variable was IPV where it combined all physical, sexual, and emotional form of violence. Series of independent questions were asked for each woman on physical, sexual and emotional violence. To identify physical violence, women were asked to confirm that whether their husband push, shake, or throw something; slap; twist arm or pull hair; punch with fist or with something else; kick, drag, or beat up; tried to choke or burn; threaten or attack with any material at them to deliberately hurt them at one point in lives. For identification of sexual violence women were asked whether their husband ever physically forced them to have sex or make other sexual acts when they do not want. Similarly, to derive emotional violence in the survey, women were asked whether their husband ever said or did something to disgrace them in the presence of others; threaten to hurt or harm them or someone close to them; or insulted or make them sense immoral about themselves [16]. In this study, indicators of specific type of spousal violence were combined to form two categories, namely women who had never experienced the specific violence, and women who had ever experienced at least one type of the specific violence. The three types of spousal violence were combined into a single spousal violence variable with binary outcomes of ever or never experienced at least one type of spousal violence.

### Explanatory variables

Based on literature, Independent variables included in the analysis are described in Table 1 [12, 13, 17–26].

**Table 1:** Description of Explanatory Variables used in the Analysis

Variable	Description	Category
Age	Age of women	15–19, 20–24, 25–29, 30–34, 35–39, 40–44, 45–49
Marital status	Marital status of women	Married, Divorced, Widowed
Education	Education level of women	Uneducated, Primary, Secondary and Higher
Religion	Religion of women	Orthodox, Muslim, Protestant, Others
Wealth Index	Wealth index of the family	Poor, Middle, Rich
TV	Frequency of watching TV	Not at all, Sometimes, Always
Radio	Frequency of listening radio	Not at all, Sometimes, Always
Residence	women Place of residence	Rural, Urban
Region	Region of women	Tigray, Afar, Amhara, Oromia, Somali, Benishangul, SNNPR, Gambela, Harari, Addis Ababa, Dire Dawa

### Method of data analysis

Based on the valid data obtained, we have performed a descriptive analysis using frequency and percentage for both dependent and independent variables. Binary logistic regression is typically used when the dependent variable is dichotomous and the independent variables are either continuous or categorical variables. One key assumption in binary logistic regression is that observations are independent of each other. Violations of the assumption of independence of observations may results in incorrect statistical inferences due to biased standard errors. After the model is fitted it is important to check how the model adequacy is, which can tell us the goodness of fit of the model. For this research we used Hosmer-Lemeshow test, which measures the correspondence between the actual and predicted values of the dependent variable [16].

Data cleaning, management and analysis were carried out using STATA, Version 12. Variables were re-coded to meet the desired classification. All hypotheses testing to determine differences, associations and relationships were judged significant at *p* < 0.05.

### Inclusion exclusion criteria

Only ever-married women in reproductive age who reported their experience of IPV were considered. Thus single/unmarried women and women who did not report their IPV experience were excluded.

## Result

In this study a total of 4714 ever-married women in reproductive age who reported their experience of spousal violence were considered from nine regional states and two city administratives. According to the result presented in Table 2, most women lived in rural areas (74.3%), but had a relatively low level of education; only 16.8% of women attended secondary and higher while half of them (49.0%) of are uneducated. The sample has a fairly young age distribution and nearly two thirds of women (71.3%) are married, while the rest of them are divorced and widowed. Over 30% of study participants were subjected to IPV. According to religion of respondents, the maximum burden of IPV is observed in other religions (37.9%) while lowest (26.3%) is observed among Muslim women.

**Table 2:** Description of Status of IPV by basic covariates

Variable	Category	Total	Domestic Violence		*P*-value
			No	Yes	
Age	15–19	1069(22.7%)	769(71.9%)	300(28.1%)	
	20–24	915(19.4%)	638(69.7%)	277(30.3%)	
	25–29	945(20.0%)	648(68.6%)	297(31.4%)	
	30–34	716(15.2%)	502(70.1%)	214(29.9%)	0.564
	35–39	507(10.8%)	349(68.8%)	158(31.2%)	
	40–44	323(6.9%)	228(70.6%)	95(29.4%)	
	45–59	239(5.0%)	165(69.0%)	74(31.0%)	
Religion	Orthodox	1778(37.7%)	1204 (67.7%)	574 (32.3%)	
	Protestant	874(18.6%)	587(67.2%)	287 (32.8%)	
	Muslin	1973(41.9%)	1454 (73.7%)	519 (26.3%)	0.000**
	Other	87(1.8%)	54 (62.1%)	33 (37.9%)	
Residence	Rural	3503(74.3%)	2422 (69.1%)	1081(30.9%)	0.002**
	Urban	1211(25.7%)	877 (72.4%)	334 (27.6%)	
Education level	Uneducated	2309(49.0%)	1656(71.7%)	653(28.3%)	
	Primary	1613(34.2%)	1099 (68.1%)	514 (31.9%)	0.082
	Secondary	539(11.4%)	368 (68.3%)	171 (31.7%)	
	Higher	253 (5.4%)	176(69.6%)	77(30.4%)	
Wealth	Poor	2108	1380 (65.5%)	728 (34.5%)	
Index	Middle	666	458 (68.8%)	208(31.2%)	0.026
	Rich	1940	1344 (69.3%)	596 (30.7%)	
Marital Status	Married	4117	2934(71.3%)	1183 (28.7%)	
	Divorced	431	243 (56.4%)	188 (43.6%)	0.000**
	Widowed	166	122 (73.5%)	44 (26.5%)	
Watching TV	Not at all	3367	2371(70.4%)	996 (29.6%)	
	≤1 a week	494	321 (65.0%)	173 (35.0%)	0.032**
	> 1 a week	853	607 (71.2%)	246 (28.8%)	
Listening Radio	Not at all	3301	2350 (71.2%)	951(28.8%)	
	≤1 a week	722	477 (66.1%)	245 (33.9%)	0.014**
	> 1 a week	691	472(68.3%)	219 (31.7%)	
Total		4714	3299(69.98%)	1415(30.02%)	

About half of the divorced women (43.6%) are victims of IPV while 26.5 and 28.7% of widowed and married women reported that they have experienced IPV respectively. The findings also showed that women who from rich and middle wealth categories are 31.2 and 30.7% respectively which is less likely to experience IPV compared to women from poor wealth category (34.5%).

Table 3 summarizes the prevalence of IPV among different regions of Ethiopia. The maximum IPV is found in Harari (39.1%) followed by Oromia and Gambela regional states 38 and 35% respectively while lowest (11.6%) is observed in Somali regional state.

**Table 3:** Summary of IPV by Regional States

Religion	N	Domestic Violence		*P*-value
		No	Yes	
Tigray	502	343 (68.3%)	159 (31.7%)	
Afar	393	309 (78.6%)	84 (21.4%)	
Amhara	569	388 (68.2%)	181 (31.8%)	
Oromia	648	402 (62%)	246 (38%)	
Somali	455	402 (88.4%)	53 (11.6)	
Benishangul Gumuz	388	257 (66.2%)	131 (33.8%)	0.000**
SNNPR	558	391 (70.1%)	167 (29.9%)	
Gambela	337	219 (65%)	118 (35%)	
Harari	281	171 (60.9%)	110 (39.1%)	
Addis Ababa	294	213 (72.4%)	81 (27.6%)	
Dire Dawa	289	204 (70.6%)	85 (29.4%)	

### Logistic regression model result

Based on the result in Table 4, poor women had greater experience of IPV (OR = 1.21; [0.9253 1.4254]) than rich women. According to place of residence, urban women have 34% less chance of experiencing IPV than rural women (OR = 0.66; CI = [0.5353, 0.8127]). The likelihood of experiencing IPV for divorced women is two times more likely than married women (OR = 1.98; CI: [1.6103, 2.4347]) while there is no significant difference between married and widowed women.

**Table 4:** Result of Logistic Regression Model

Variable	Category	OR	95% CI for OR	*P*-Value
Wealth Index	Rich	Ref		
	Middle	1.04	[0.86 1.26]	0.6890
	Poor	1.21	[1.07 1.43]	0.0325*
Marital status	Married	Ref.		
	Divorced	1.98	[1.61 2.43]	0.000*
	Widowed	0.913	[0.64 1.30]	0.6150
Place of residence	Rural	Ref.		
	Urban	0 .66	[0.54 0.81]	0.000*
	Uneducated	Ref.		
Education level	Primary	1.32	[1.12 1.55]	0.0010*
	Secondary	1.34	[1.05 1.70]	0.0200*
	Higher	1.20	[0.86 1.65]	0.8610
	15–19	Ref.		
	20–24	1.19	[0.97 1.45]	0.094 0
Age	25–29	1.32	[1.08 1.62]	0.0080*
	30–34	1.30	[1.04 1.63]	0.0210*
	35–39	1.36	[1.06 1.74]	0.0150*
	40–44	1.21	[0.91 1.61]	0.2000
	45–49	1.38	[0.10 1.90]	0.0510

According to women’s level of education, women having primary and secondary education level are 1.32 and 1.34 times more likely to experience IPV than uneducated women respectively while there is no significant difference between women having higher education and uneducated women (*p*-value = 0.861). The odds of affected by IPV for women 25–29, 30–34, 35–39 years old are 1.32 (CI: [1.0752, 1.6150]), 1.30 (CI: [1.0409, 1.6337]), 1.36 (CI: 1.0616, 1.7418]) times more likely than women 15–19 years old.

Hosmer and Lemeshow test (Table 5) revealed that *P*-value = 0.596 indicating the model fits the data very well. This is good and implied that the model is indeed correctly specified.

**Table 5:** Hosmer and Lemeshow Test

Chis-square	Df	Sig.
1.631	8	0.596

## Discussion

Women are vulnerable to violence from many different sources, but the WHO multi-country study on women’s health and domestic violence against women of 2005 said that most violence against women is executed by an intimate partner [5]. The main goal of this study was to assess the prevalence of IPV against ever-married women and associated factors in Ethiopia. Accordingly, the result showed that nearly one in three ever-married women in Ethiopia (30.2%) faced at least one type of IPV which indicates considerable number of women in the country is still suffering from it. These findings are consistent with a literature that shows IPV is common in Africa [5, 17].

The prevalence of IPV against women in this study is comparable with the result of other similar studies in Ivory Coast (32.1%) [18], KwaZulu-Natal, Turkey (30.0%) [27] and South Africa (31%) [28]. This result was also revealed that the prevalence of IPV is decreased by more than half from the finding of WHO multi-country study where the prevalence was 71%. This difference may be due to the improvement made on IPV for the last 11 years in the Ethiopia. Furthermore, this multi-country study used a single rural setting from Ethiopia where intimate violence is estimated to be high.

In addition, the government is working hard on empowering female and avoiding dragging factors to increase political, social, and economical contribution of women for their country. Moreover, the prevalence seen in this study was lower than those of studies conducted in countries such as Bolivia (47%), Southern Sweden (39.5%), Ghana (39%), Portuguese (43.4%), and Pakistan (51%) [19, 29–32].

The study also revealed that divorced women are more likely to experience IPV than married women while there is no significant difference between married and widowed women. This high prevalence of IPV is expected to be the reason to be divorced. Similar results have been found in research conducted in Arkansas and New Mexico [21] and researchers also found that divorced Canadian women are four times more likely to be abused by a previous partner than married women [22].

The results of this study suggested that place of residences was significant predictive factor for IPV. Urban women have less chance of experiencing IPV than rural women. This higher prevalence rate of IPV on rural women may be due to the cultural perception, lack of knowledge and information in rural society where beating, insulting and other form of violence is considered as a means of shaping wife’s behavior. Consistent results were also observed in other research such as in Nigeria [12], southeast Nigeria [20], they reported that rural women had greater chance of facing IPV. But findings from cross-sectional household surveys in eight southern African countries [17] showed that there is no significant difference on IPV among rural and urban women.

This study also showed that the likelihood of experiencing IPV increases with maternal education but there is no significant difference with women having higher education. This finding is consistent with research conducted in Nigeria [12]. But Contrary result was obtained in research conducted in Ivory Cost [18], Nigeria [12] where the likelihood of experiencing spousal violence reduces as maternal education improves. The study also revealed that women 25–39 years old are more likely to experience IPV than 15–19 years old women. Similar result has been found in research conducted in eight southern African countries [17].

### Strength and limitation

The primary strength of this study is that the analysis was based on the data collected from all regions of the country. Relatively large sample size respondents have been selected randomly and the data was collected by well-trained data collectors with strong supervision to maximize data quality. As limitation, this study used cross-sectional data that has limitation to determine causality. Furthermore, IPV is a sensitive subject that may be associated with negative feelings of guilt and stigma. Consequently, the women may have been reluctant to disclose their experiences of intimate partner violence, which may have affected the reported prevalence in this study. Thus, the findings of this study should be interpreted within this limitation.

## Conclusion

Despite the fact that the government tried to lower violence against women, the prevalence of IPV among ever married women is high in Ethiopia. A total of 4714 ever married women in the reproductive age were considered in this study from which about 30% of them exposed to IPV. Based on these results, living in rural, being poor, being divorced and being 25–39 years old are found to be significant predictors of IPV. Hence, improving economic status of household and awareness creation for rural resident can be effective strategies to reduce IPV.

## Data Availability

The dataset was demanded and retrieved from CSA website after formal online registration and submission of the project title and detail project description. The data can be accessed through http://www.statsethiopia.gov.et/.

## Abbreviations

CI: Confidence interval
CSA: Central Statistical Agency
Df: Degree of freedom
EAs: Enumeration Areas
EDHS: Ethiopian Demographic and Health Survey
IPV: Intimate partner violence
OR: Odds Ratio
WHO: World Health Organization
